# Supplementary material for: A CRISPR screen identifies genes controlling Etv2 threshold expression in murine hemangiogenic fate commitment
Source: Nat Commun. 2017 Sep 14;8:541. doi: 10.1038/s41467-017-00667-5 (PMC5599515; doi:10.1038/s41467-017-00667-5)
Supplement: Supplementary file 1 — Supplementary Information [file 41467_2017_667_MOESM1_ESM.pdf]

Description of Supplementary Files

File Name: Peer Review File

File Name: Supplementary Information
Description: Supplementary Figures, Supplementary Table, Supplementary References.

File Name: Supplementary Data 1
Description: Expression of 73 ETV2 target genes in 4 populations from SGET cell differentiation

File Name: Supplementary Data 2
Description: Transcriptome analysis of ES cell mesoderm differentiation and hemangiogenesis

File Name: Supplementary Data 3
Description: "pr"-enriched genes in Goode's data

File Name: Supplementary Data 4
Description: "dp"-enriched genes in Goode's data

File Name: Supplementary Data 5
Description: Transcription-related genes for comparison

File Name: Supplementary Data 6
Description: All the reads counting data for screen

File Name: Supplementary Data 7
Description: Genes important for ES cell viability or maintenance

File Name: Supplementary Data 8
Description: Genes implicated in mesoderm differentiation

File Name: Supplementary Data 9
Description: CRISPR screen for genes implicated in Etv2high cell generation and chosen candidates

File Name: Supplementary Data 10
Description: 215 genes that are expressed in mesoderm and rank in top500 for depletion from pr cells to Etv2high cells

File Name: Supplementary Data 11
Description: RNA-seq data for ΔFoxh1-pr cell

File Name: Supplementary Data 12
Description: Transcription-related genes for all samples including ΔFoxh1-pr cell

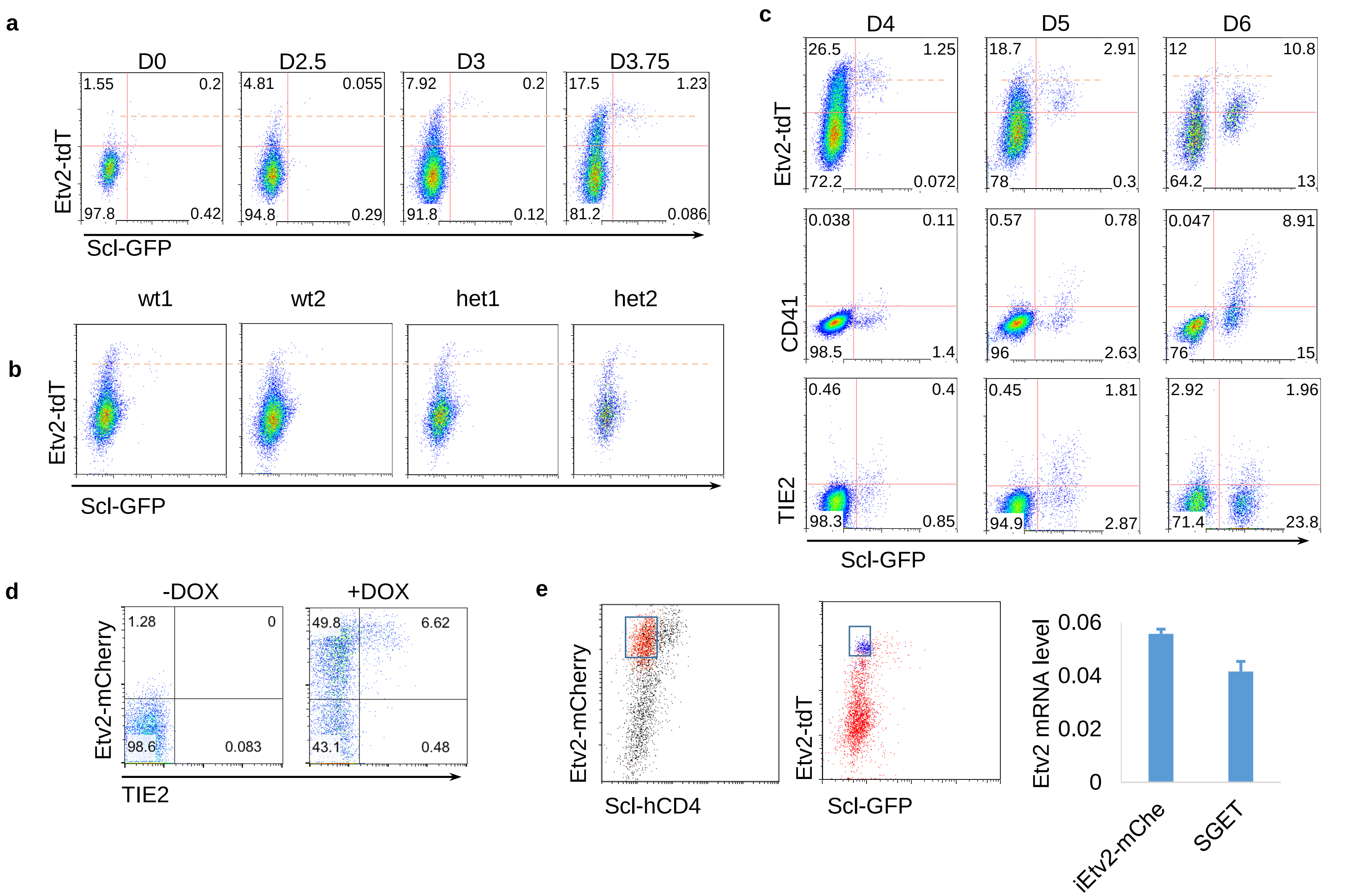

Supplementary Figure 1. Threshold of Etv2 in activating hemangiogenic genes (related to Figure 1) (**a**) Dynamics of Etv2-tdTomato and Scl-GFP expression in SGET ES cell differentiation (D0-3.75) was examined by flow cytometry. (**b**) Flow cytometry analysis of D4 EBs of wild type (wt) and Etv2-het(erozygote) SGET ES cell clones. (**c**) Dynamics of CD41 and TIE2 expression in comparison to Etv2-tdTomato and Scl-GFP expression in D4-6 SGET EBs is shown. (**d**) Expression of TIE2, another ETV2 target gene, is dependent on *Etv2* threshold expression. Flow cytometry analysis of Etv2-mCherry and TIE2 of D3.5 iEtv2-mCherry EBs differentiated in serum free conditions. -DOX, no DOX control; +DOX, DOX was added at 2µg/mL from D2.5-3.5. (**e**) Relative Etv2 mRNA levels in Etv2-mCherry^{hi}Scl-GFP^{-} and Etv2-tdTomato^{hi}Scl-GFP^{-} cells (marked as colored populations in boxes) sorted from D3.5 iEtv2-mCherry EBs and D4 SGET EBs, respectively. DOX (2µg/mL) was added from D2.5-3.5 for Etv2-mCherry induction. Error bars are s.d..

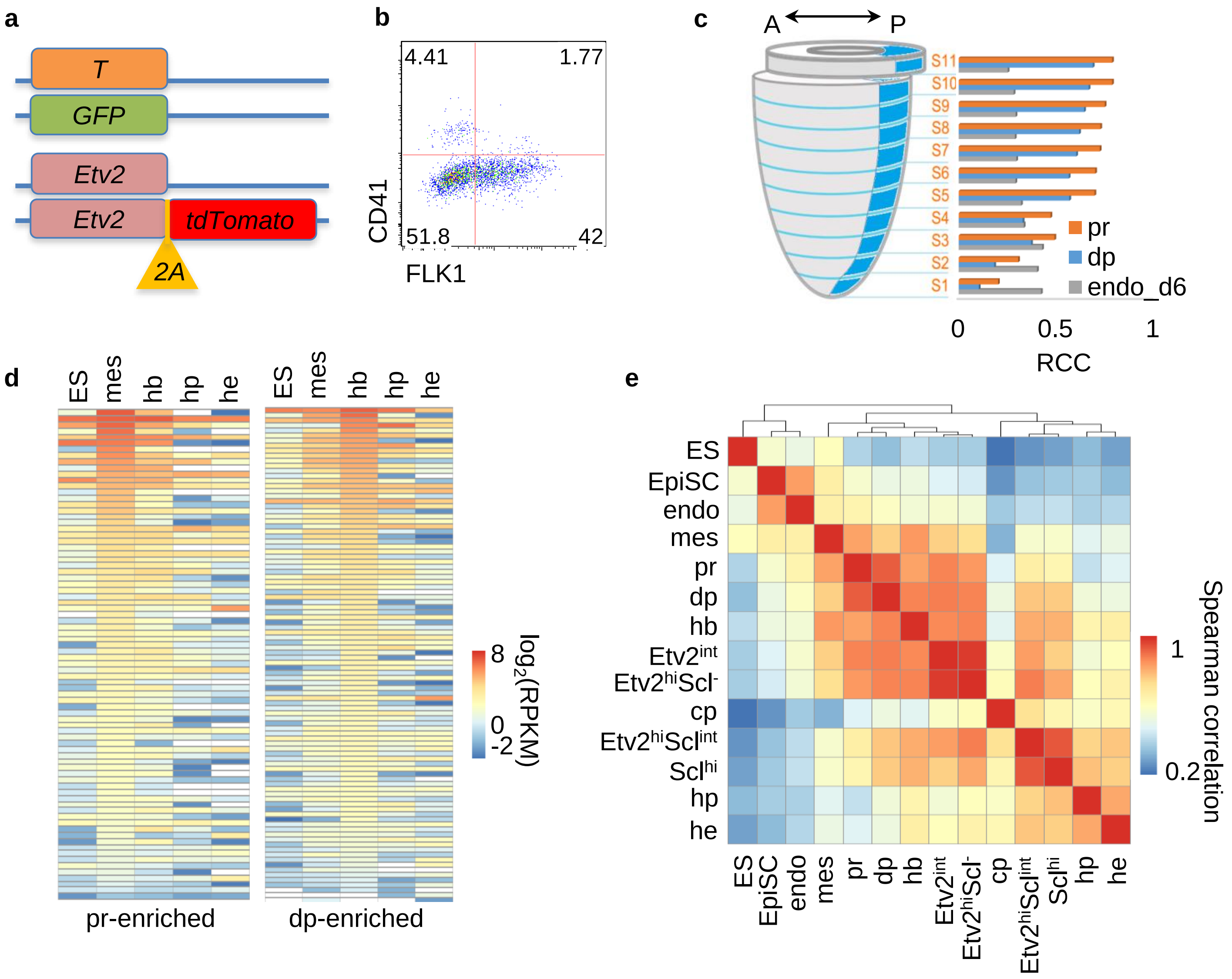

Supplementary Figure 2 Developmental route of Etv2^{hi} hemangiogenic progenitors (related to Figure 2) (**a**) Scheme of the TGET reporter ES cell line. (**b**) Sorted PDGFRa single positive cells from D3.5 TGET EBs were reseeded onto OP9 stromal cells and cultured for additional 4 days, and subjected to flow cytometry analysis for CD41 and FLK1. (**c**) Transcriptomes of "pr", "dp", and "endo_d6" cells was compared to those of 11 tandem regions along the primitive streak of E7.0 mouse gastrulating embryo, using Zipcode mapping[1]. The bars show the Spearman rank correlation coefficients between the transcriptomes of "pr", "dp", or "endo_d6" vs. a specific region of primitive streak (see Supplementary Methods). Endo_d6, ES cell-derived D6 endoderm differentiation cells, was used as a control, which showed closer correlation to the distal regions of the primitive streak. (**d**) Expression patterns of "pr" (left, Group III in Fig. 2h and Supplementary Table 2) and "dp" (right, Group IV in Fig. 2h and Supplementary Table 2)-enriched genes in a published RNA-seq analysis of mesoderm differentiating ES cells[2]. (**e**) Spearman's rank correlation of transcription factor and transcription-related gene expression among indicated samples.

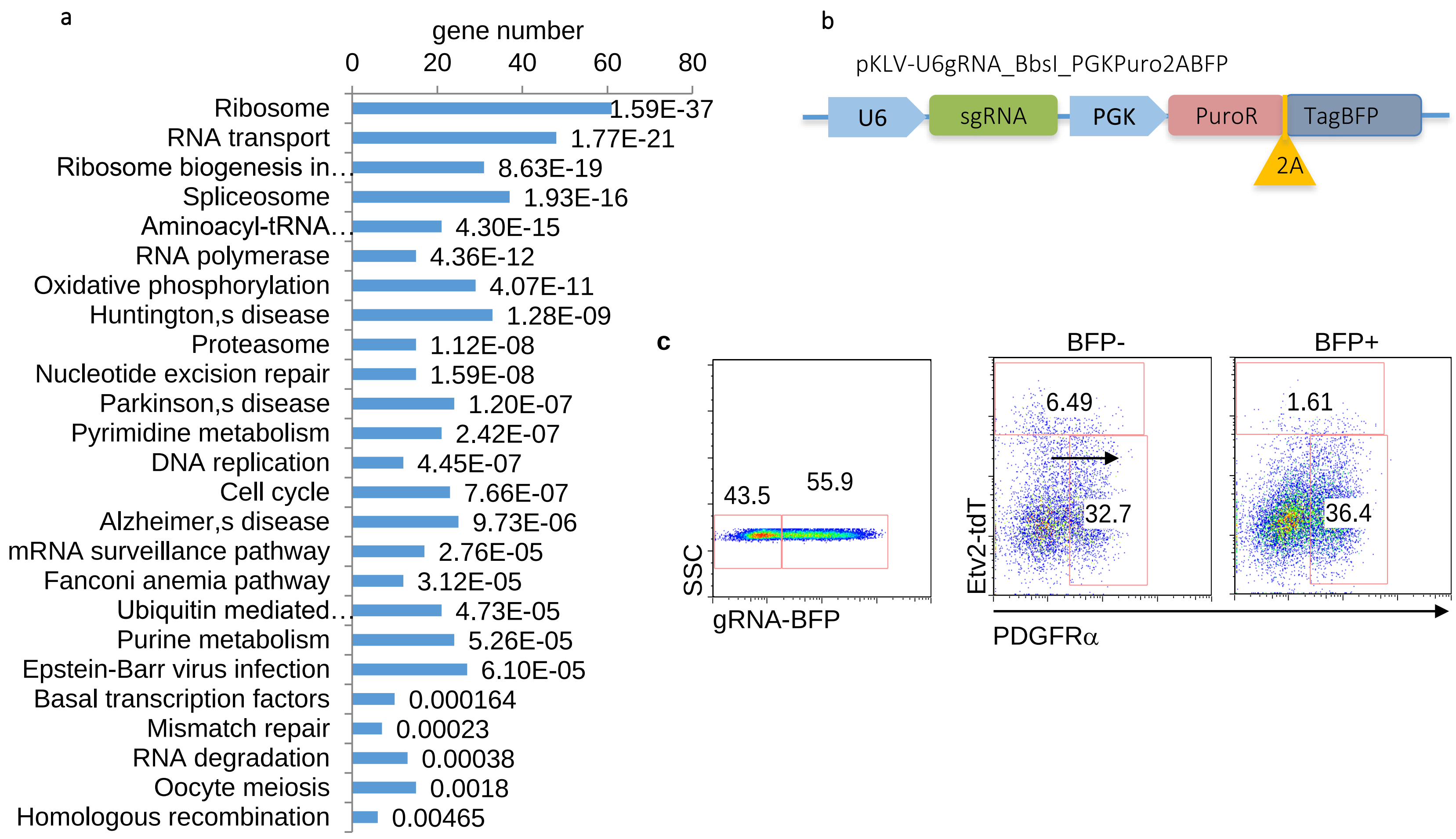

Supplementary Figure 3. CRISPR screening identifies key *Etv2* upstream signals (related to Figure 3) (**a**) Functional annotation of pathways with genes depleted from ES cells to PDGFRα^{+} cells. (**b**) Scheme of the lentiviral construct expressing sgRNA and BFP used for validation of chosen candidates. (**c**) An example used for a candidate sgRNA validation: BFP^{-} (non sgRNA-expressing) or BFP^{+} (sgRNA-expressing) cells were used to compare the ratio of Etv2^{high} to PDGFRα^{+}. Cells successfully infected with the lentivirus expressing sgRNA against a specific gene simultaneously express the blue fluorescent protein BFP, which can be identified by flow cytometry.

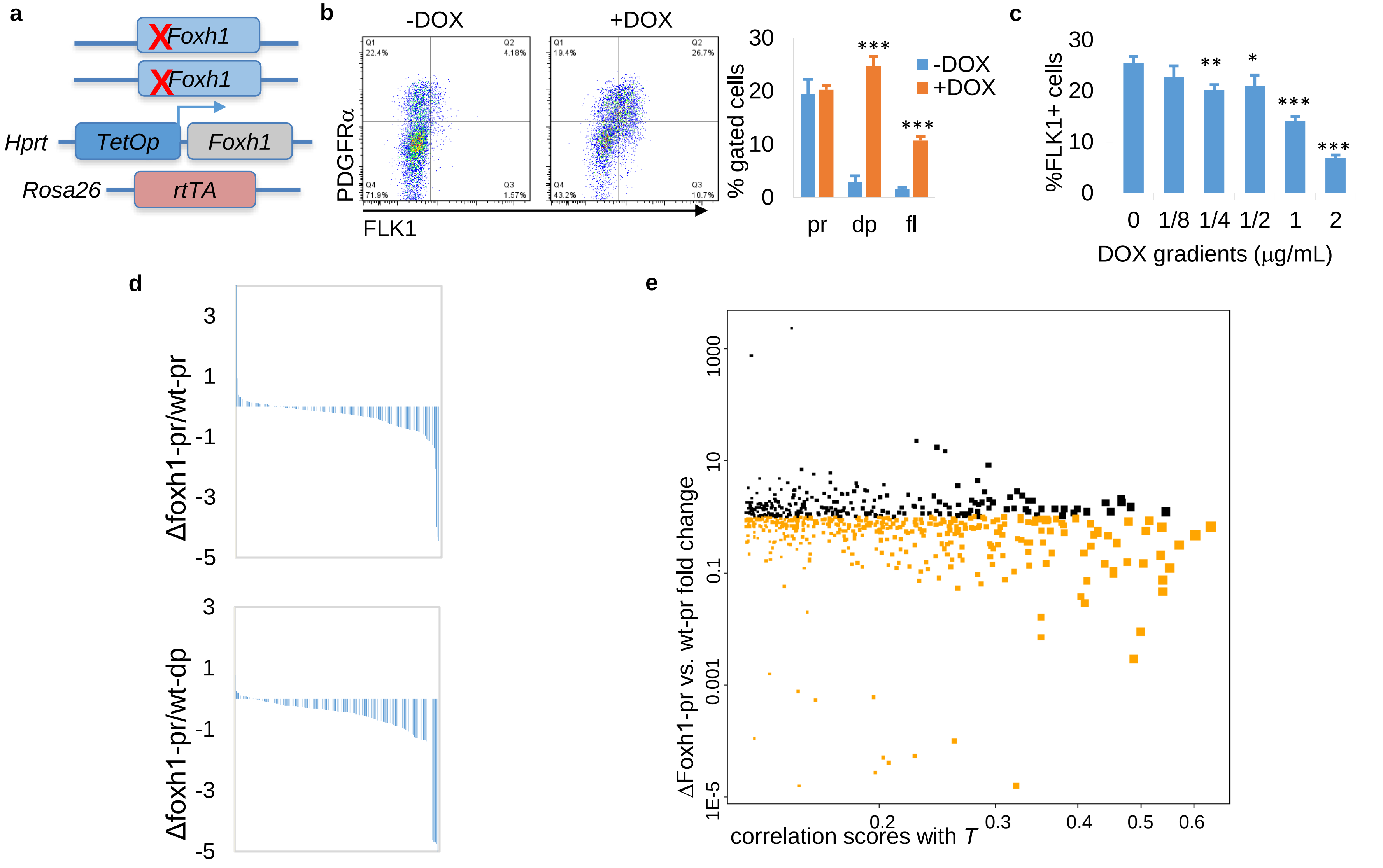

Supplementary Figure 4. Foxh1 is required for FLK1$^+$ mesoderm generation (related to Figure 4) (**a**) Scheme of the *iFoxh1-ΔFoxh1* ES cell line. (**b**) FLK1$^+$ cells can be rescued by exogenous *Foxh1* expression from *ΔFoxh1* ES cells. iFoxh1-ΔFoxh1 ES cells were differentiated in the presence or absence of DOX and analyzed for PDGFRα and FLK1 on D3. DOX was added at the beginning of differentiation at 500ng/mL. (right) The percentage of PDGFRα^+FLK1$^-$ ("pr"), PDGFRα^+FLK1$^+$ ("dp") and PDGFRα^-FLK1$^+$ ("fl") generated in the presence or absence of DOX is shown. (**c**) iFoxh1-wt ES cells were differentiated with different concentrations of DOX (added on D0), and FLK1$^+$ (including "dp" and "fl") cells were analyzed on D3. The percentage of FLK1$^+$ cells generated at different concentrations of DOX is shown. (**d**) (top) Bar graphs showing log2-transformed fold changes of "pr" (Group III in Fig. 2h)-enriched genes in RNA-seq data (*ΔFoxh1*-pr vs. wt-pr); (bottom) log2-transformed fold changes of "dp" (Group IV in Fig. 2h)-enriched genes in RNA-seq data (*ΔFoxh1*-pr vs. wt-dp). (e) x-axis showing the gene's correlation score to *T* for gastrulation initiation in early embryo, y-axis showing the gene's fold change comparing RNA-seq RPKMs of *ΔFoxh1*-"pr" cells to that of wt-"pr" cells. Downregulated genes in *ΔFoxh1*-"pr" cells were shown in yellow, otherwise in black. *, P-value<0.05 in Student's t-Test, **, P<0.01, ***, P<0.001, and n=3. Error bars are s.d..

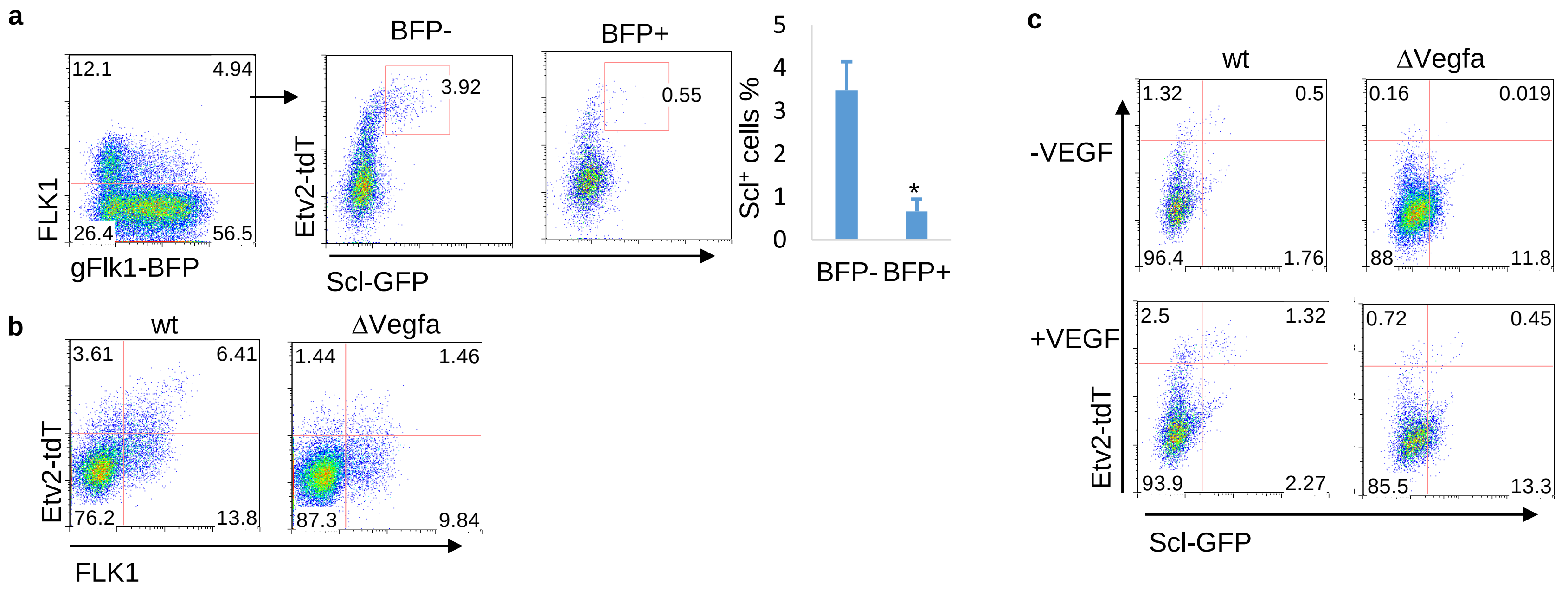

Supplementary Figure 5. VEGF signaling is essential for *Etv2* threshold expression (related to Figure 6) (**a**) Flow cytometry analysis of D4 wt SGET EB cells infected with lentivirus that expresses BFP and sgRNA against *Flk1*. The percentage of Scl-GFP^{+} cells within BFP^{-} (wt) and BFP^{+} (Δ*Flk1*) cells is shown on the right. (**b**) Flow cytometry analysis of Etv2-tdTomato and FLK1 expression in D5 wt and Δ*Vegfa* SGET EB cells in serum free conditions supplemented with BMP4 (5ng/mL). (**c**) Flow cytometry analysis for Etv2-tdTomato and Scl-GFP of D5 Δ*Vegfa* SGET EB cells differentiated in serum free conditions supplemented with BMP4 (5ng/mL) and ±VEGF-A (25ng/mL) from the beginning of differentiation. *, P-value<0.05 in Student's t-Test, n=3. Error bars are s.d..

Supplementary References

1. Peng, G., *et al.* Spatial Transcriptome for the Molecular Annotation of Lineage Fates and Cell Identity in Mid-gastrula Mouse Embryo. *Dev Cell* **36**, 681-697 (2016).
2. Goode, D.K., *et al.* Dynamic Gene Regulatory Networks Drive Hematopoietic Specification and Differentiation. *Dev Cell* **36**, 572-587 (2016).

Supplementary Table 1. A list of primers and sgRNAs used in this work

sgRNAs	Etv2 tdTomato knockin	ttttattggccttctgcacc
	Foxh1 knockout	cttatggaagcaccgattag
	Flk1 knockout	gtcccggtacgagcacttgt
	Vegfa knockout	ttctcgctccgtagtagccg
	Etv2 single allele knockout/het	atcacaccaatgaacgtaga

primers for Etv2-tdTomato knockin clone identification	Upstream of 5'-arm	ACAAGCTGACAGGACTGG
	Inside tdTomato	TGGTGTAGTCCTCGTTGTGG
	Inside PGK-Hyg cassette	CACGATTGTCATGCCCACGC
	Downstream of 3'-arm	CACGCACTGTGGAAACAAGGAC
primers for RT-qPCR	Eomes-f	GGCCCCTATGGCTCAAATTCC
	Eomes-r	CCTGCCCTGTTTGGTGATG
	Ets1-f	ACAGACTACTTTCGGATCAAGCA
	Ets1-r	ACGCTCTCAAAAGAGTCCTGG
	Etv2-f	ACAGCTACATTTTCAAGGCC
	Etv2-r	GTCCGAGGTGTTGCATCCC
	Evx1-f	GAGTACCAGCACAGCAAAGC
	Evx1-r	CTGCCACCGTTACTCTTGGG
	Flk1-f	TCCATCTTTTGGTGGAATGA
	Flk1-r	CTGGAGTACACGGTGGTGTC
	Foxa2-f	CCCTACGCCAACATGAACTCG
	Foxa2-r	GTTCTGCCGGTAGAAAGGGA
	Foxh1-f	TTATCCGTCAGGTCCAGGCA
	Foxh1-r	TCAGCAGGAATCAGGCTCAC
	Frzb-f	CACAGCACCCAGGCTAACG
	Frzb-r	TGCGTACATTGCACAGAGGAA
	Gsc-f	CAGATGCTGCCCTACATGAAC
	Gsc-r	TCTGGGTACTTCGTCTCCTGG
	Hand1-f	GGCAGCTACGCACATCATCA
	Hand1-r	CCTGGCATCGGGACCATAG
	Lmo1-f	GAGGCTTTTTGGCACCACAG
	Lmo1-r	TCTCTGATTGCAGAGCTGGC
	Med4-f	TGGAGGTCTTGTCGAGGGAA
	Med4-r	CTTGGAAGTCCCCATCTCGG
	Mesp1-f	GTCACTCGGTCCTGGTTTAAG
	Mesp1-r	ACGATGGGTCCCACGATTCT
	Mixl1-f	ACGCAGTGCTTTCCAAACC
	Mixl1-r	CCCGCAAGTGGATGTCTGG
	Oct4-f	AGTGGGGCGGTTTTGAGTAA
	Oct4-r	GGTGTACCCCAAGGTGATCC
	Pbx3-f	GGACATCGGCGACATCCTC
	Pbx3-r	CGCCGGTTTCATTCTGTGAC
	Scl-f	GGGCTTTTGGCGATAGGTC
	Scl-r	GGAATCTCCACACCCAGACAG
	Six2-f	AGGCCAAGGAAAGGGAGAAC
	Six2-r	GAACTGCCTAGCACCGACTT
	Smad1-f	GCTTCGTGAAGGGTTGGGG
	Smad1-r	CGGATGAAATAGGATTGTGGGG
	T/Brachyury-f	GGTCTCGGGAAAGCAGTGGC
	T/Brachyury-r	CATGTACTCTTTCTTGCTGG
	Tbx3-f	GAACCTACCTGTTCCCGGAAA
	Tbx3-r	CAATGCCCAATGTCTCGAAAAC
	Tlx2-f	CAACCTCTCAGGCTTGGACC
	Tlx2-r	TCTGATCGATGCCGAAGCTG
	Trib1-f	TCCGAAGCCTCCTAAGACGA
	Trib1-r	GTCAACATAGCCCGGTTCCA
	Vax1-f	CCAGGGTCTCGAAGAACGC
	Vax1-r	ACAATCTTCCGAAGCGCCAG
	Zcchc12-f	TGCCTTGGAAACTGCGCTAT
	Zcchc12-r	CTCTACAACCAGCTGCACCA
